# Sensitive Five-Fold Local Symmetry to Kinetic Energy of Depositing Atoms in Cu-Zr Thin Film Growth

**DOI:** 10.3390/ma11122548

**Published:** 2018-12-14

**Authors:** Lu Xie, Haojie An, Qing Peng, Qin Qin, Yong Zhang

**Affiliations:** 1 School of Mechanical Engineering, University of Science and Technology Beijing, Beijing 100083, China; anhaojie@xs.ustb.edu.cn (H.A.); qinqin@me.ustb.edu.cn (Q.Q.); 2 Nuclear Engineering and Radiological Sciences University of Michigan, Ann Arbor, MI 48108, USA; 3 State Key Laboratory for Advanced Metals and Materials, University of Science and Technology Beijing, Beijing 100083, China; drzhangy@ustb.edu.cn

**Keywords:** molecular dynamics simulation, metallic glasses, glass forming ability, thin film growth, five-fold local symmetry

## Abstract

We have investigated the glass formation ability of Cu-Zr alloy by molecular dynamics simulation of the deposition process. The atomistic structures of Zr_x_Cu_100−x_ metallic glass films have been systematically examined under the growth conditions of hypereutectic-eutectic, near-eutectic, and hypoeutectic regions by the radial distribution function and simulated X-ray diffraction. The structure analysis using Voronoi polyhedron index method demonstrates the variations of short-range order and five-fold local symmetry in Zr_x_Cu_100−x_ metallic glass films with respect to the growth conditions. We manifest that the five-fold local symmetry is sensitive to the kinetic energy of the depositing atoms. There is positive correlation between the degree of five-fold local symmetry and glass forming ability. Our results suggest that sputtering conditions greatly affect the local atomic structures and consequential properties. The glass forming ability could be scaled by the degree of five-fold local symmetry. Our study might be useful in optimizing sputtering conditions in real experiments, as well as promising implications in material design of advanced glassy materials.

## 1. Introduction

A solid metal in general is a crystalline where atoms have long range orders. When they are quenched with ultra-high cooling rate from melt, they form amorphous metals, namely metal glasses (MGs). Unlike common glasses, metallic glasses are metals, and therefore good in electrical conductivity and thermal conductivity. Without long-range orders or translational symmetry in structure, metallic glasses in general possess much higher strength, hardness, and corrosion resistance than their crystalline counterparts [1,2,3,4]. As a result, MGs have attracted extensive interest due to their exotic mechanical properties and promising applications [5,6]. However, the poor glass-forming ability (GFA) limits the broad engineering application of MGs, which restricts both the shape and the size of MGs. The key to the design of MGs with great GFA is to understand the structural origin of GFA, which, however, is a long-standing challenge.

Metallic glasses are usually obtained by rapid cooling of multicomponent alloy that the crystallization is avoided. The binary Zr-Cu alloy is a paradigm system to study the atomistic structures properties relationships and glass transition due to the outstanding glass formation ability which originated from the ideal atomic size ratio between the two constituents [7]. Recent studies have shown that binary Zr-Cu alloys have the ability to form a variety of stable metallic glass with different atom ratios [8,9], and the amorphous film formed by Zr-Cu alloys has prefect mechanical [10,11,12] and superconducting properties [13].

Currently, MGs thin films are mainly prepared by magnetron sputtering deposition method. There are lots of experiments and theoretical studies on this deposition process. For example, Dudonis et al. [14] performed electron bombardment of Cu and Zr targets at different powers to prepare different compositional Zr_x_Cu_100−x_ films (5 ≤ x ≤ 95) in a magnetron sputtering deposition. Apreutesei et al. [15] prepared Zr-Cu MG films by DC magnetron sputtering in argon gas, and studied the influence of deposition parameters on structure and elastic properties. Daisman et al. [16] used single-target magnetron sputtering method to prepare Zr-based MG thin films with higher stability. Xu et al. [17] studied the correlation between GFA and mechanical behavior of Zr-Cu MGs. It is intriguing to see if there exists a similar correlation between GFA and microstructure.

Besides experiments, the method of molecular dynamics simulations (MD simulation) has been well established in the past decades and becomes a reliable and indispensable tool in various investigations [18]. For MGs, Utz et al. [19] introduced an algorithm for plastic deformation of A_80_B_20_ two-component metallic glasses, which allows the use of MD simulations to study the volume changes related to plastic deformation directly. Sha et al. [20] studied the medium/short-range order of Zr_64_Cu_36_ MG by using MD method. The compressive mechanical properties of Zr_85_Cu_15_ nanocrystal-MG composites have been analyzed via MD simulations [21]. Yang et al. [22] studied the size effects in Zr_50_Cu_50_ MG films via MD simulations. In our previous work [23], we use the MD method to simulate the deposition process of Zr_x_Cu_100−x_ metallic glass film and investigated the effect of different element ratios.

The microstructure and glass forming mechanism are among the main important topics of MD studies. Sha et al. [20,24] studied the forming conditions of Zr-Cu MGs by using MD simulation. Hajlaoui et al. [25] observed the microstructure of Zr-Cu MGs during deformation by in-situ HRTEM observation. The studies from Almyras et al. [26] show that both Zr_35_Cu_65_ and Zr_65_Cu_35_ MGs are composed of supercluster formed by icosahedral-like clusters. Tripathi et al. [27] proposed a glass forming ability expression that does not use the characteristic temperature, which analyzes the GFA of the alloy from the component data. Turchanin et al. [28] considered that the interaction between components is an important reason for amorphization of alloy through the study of enthalpy of Zr-Cu system. The theory proposed by Zhang et al. [29] claims that the formation of the microstructure of the multi-alloy has a direct relationship with the property parameters Ω and δ (Ω is the ratio of average melting temperature timing the mixing entropy and the mixing enthalpy, and δ is the mean square deviation of the atomic size), which can be used to predict whether the alloy can form metallic glass. It was reported that five-fold local symmetry (FFLS) is an important characteristic in MGs. Hu et al. [30] studied the structure evolution in metallic glass-forming liquids by FFLS. Lu et al. [31] studied the correlation between FFLS and dynamical slowing down in Cu-Zr glass-forming liquid, and results show that FFLS has a close relation to GFA.

For the Zr-Cu system, there are few studies on its crystal-amorphous transition and the relationship between microstructure and GFA, while current research only involves the deposition process and the mechanical properties of the metal film. The metallic glass transition mechanism remains elusive. With the well-established molecular dynamics simulations, we can accurately simulate the deposition process of Zr-Cu alloy thin films and explore the film evolution process at the atomic level. Therefore, MD method is employed in this study. We simulate the deposition process of amorphous films of Zr_x_Cu_100−x_ (x = 50, 70, 90) in sub-eutectic, near-eutectic, and hypoeutectic regions. The crystal-amorphous transition has been studied in depth. Its glass forming mechanism and the relationship between microstructure and GFA have been analyzed upon these simulations.

## 2. Models and Methods

All MD simulations in this work were performed using the Large-scale Atomic/Molecular Massively Parallel Simulator (LAMMPS) package (Sandia, Albuquerque, NM, USA) [32]. The structural analysis and post-processing were performed with OVITO [33].

### 2.1. Modeling

**Figure 1:**
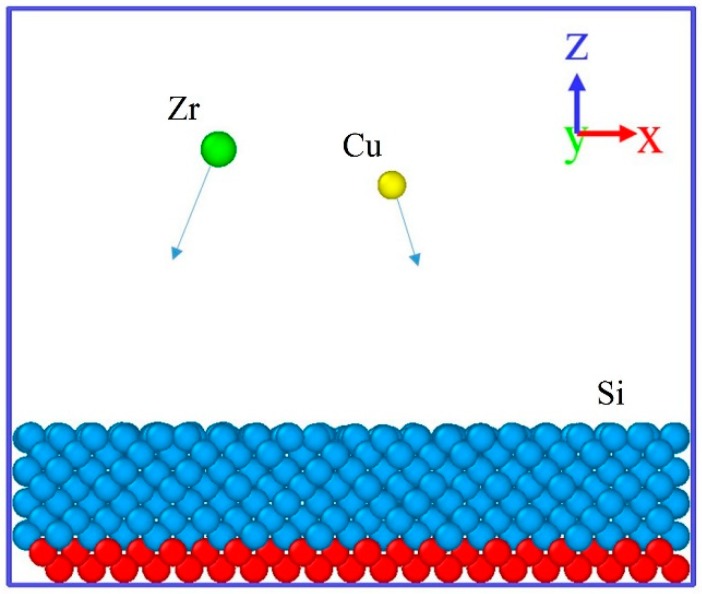
Schematic picture of the deposition model. Green atom is Zr, yellow atom is Cu, blue and red atoms are moving and fixed Si, respectively. The simulation box is periodic in the x and y directions. (In order to facilitate the display of nanostructures, the atom radius in the figure does not represent the actual atomic size, the same below).

Figure 1 shows the initial deposition model used in this work. Each simulation box has periodic boundary conditions only in x and y directions. Si (1 0 0) with fixed bottom atoms was used as the substrate and the dimensions were (25 × 25 × 10) Å^3^. In the deposited process, two kind of deposited atoms were generated randomly. Both Zr and Cu atoms deposited from an initial position 5–7 Å above the deposition model.

For all simulations, the microcanonical ensemble (NVE) was employed with a Berendsen thermostat [34]. Berendsen thermostat can effectively dissipate the high energy coming from Zr-Cu atoms. The temperature of the simulation box was controlled to 300 K, which is similar to experimental temperature condition.

In these simulations, embedded atom method (EAM) many-body potential [35] was applied to describe the atomic interactions of the Zr-Cu systems. The interactions between Si and Si atoms were described by Tersoff empirical potential [36]. Lennard Jones potential with Lorenz Berthelot mixing rules [37] was employed for the atomic interactions between Si and Zr-Cu systems.

### 2.2. Simulation Process

We use the modified Thompson formula (detailed in Supplementary) to calculate the mean kinetic energy [38] of the incoming Zr and Cu atoms. We examine three different experimental conditions (Table 1). The velocities of the incoming atoms are randomly sampled from a Maxwell Boltzmann distribution. The aim is to simulate the three different conditions in metallic glass growth, and to gain insights of the effect of both the incoming atom kinetic energy and the composition on the expected phase formation of Zr-Cu system.

The deposited atoms are randomly generated. The initial velocity and direction are given to make them continuously deposited to the substrate. The time step is chosen to be 0.001 ps for the compromise of computation speed and numerical instability. Every 2 ps, 5 atom are released toward the surface. This rate of 0.5 atom/ps is carefully selected so that there is enough time for thermal relaxation to take place with Berendsen thermostat.

For post-simulation analysis, we use the radial distribution functions (RDF) to determine the correlation between the atoms. The simulated X-ray diffraction (XRD) was employed to study the phase of Zr-Cu film and the Voronoi polyhedron index method (VPIM) [39] was employed to analyze the local atomic structure and FFLS of the system.

## 3. Results and Discussion

In order to study the deposition process and structural evolution of Zr-Cu alloy, MD simulations of the growth of Zr_x_Cu_100−x_ alloy and x is percentage of the atomic concentrations. We investigate x = 50, 70, 90, which is response to the sub-eutectic, near-eutectic, and hypoeutectic composition, respectively. The growth of the alloys was performed in three different energies, as listed in Table 1, with initial conditions similar to the experimental operating ones.

### 3.1. Zr_50_Cu_50_

**Figure 2:**
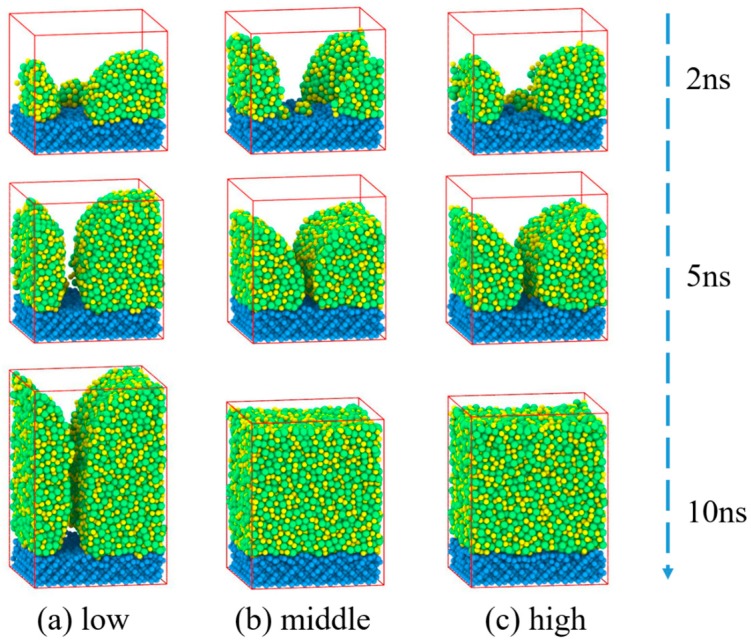
Snapshots of Zr_50_Cu_50_ deposited on Si substrate at 2 ns, 5 ns and 10 ns under different energies. The (**a**), (**b**), and (**c**) show the deposition process under three different energies, and the films grew to approximately 7.0 nm, 5.5 nm, and 5.5 nm thickness respectively.

**T**he Zr_50_Cu_50_ deposition processes with different conditions on the Si substrate are shown in Figure 2. The results show that as the mean kinetic energies increases, the morphology goes through three different stages: cluster–film–film. For Zr_50_Cu_50_ the Zr and Cu atoms grow as a cluster with height 7.0 nm under low energy. Under middle and high energies, the deposited atoms grow as clusters before 5 ns. Finally, two 5.5 nm thickness films were formed at middle and high energies. The mean kinetic energy of the deposited atoms is relatively lower at low energy (E_Zr_ = 0.13 eV, E_Cu_ = 0.34 eV), which is less than the activation energy for atomic diffusion. Therefore, the atoms are easier to aggregate with each other to form clusters.

**Figure 3:**
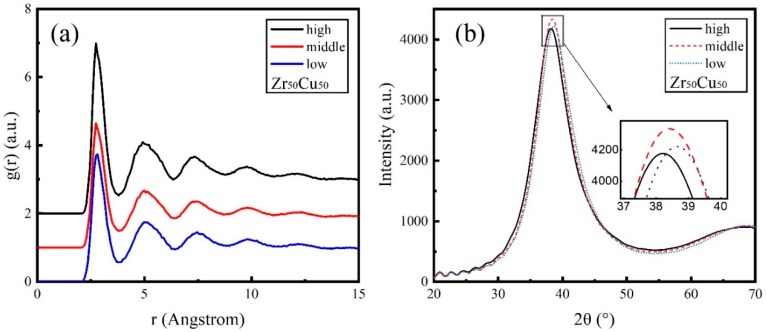
Simulated deposition of Zr_50_Cu_50_ thin films. (**a**) Radial distribution function g(r) of deposited alloy film under different energies, and the abscissa indicates atomic distance r; (**b**) the relationship between X-ray diffraction intensity and diffraction angle 2θ of deposited alloy film under different energies.

The total RDFs for Zr_50_Cu_50_ under three energies are presented in Figure 3a. For MGs, the RDF peak positions are the inherited characteristic constant sequence [40] and the peaks show the nearest neighbor distance of atoms, which can be compared with the experimental values of the two elementals (Supplementary Table S1). The RDF shows an asymmetrical first peak and a second peak with no spilt in three energies, implying that there exists a strong short-range order. While the shoulder peak on the right side of the first peak in deposited Zr_50_Cu_50_ films are not observed as literature [41]. The three energies in our simulations show that the RDF distributions are similar, and no effect of the mean kinetic energy is observed for Zr_50_Cu_50_.

The XRD patterns for Zr_50_Cu_50_ are illustrated in Figure 3b as a function of the mean kinetic energy. All of these patterns show broad diffraction peaks without any detectable sharp Bragg peaks, implying a low order structure. Zr_50_Cu_50_ films show an amorphous XRD pattern which is consistent with the calculated RDF in Figure 3a. As the mean kinetic energies increases, the value of the first peak in the XRD pattern gradually decreases. The diffraction angles of the first XRD peaks for Zr_50_Cu_50_ under three energies are between 38 and 39 degrees.

### 3.2. Zr_70_Cu_30_

**Figure 4:**
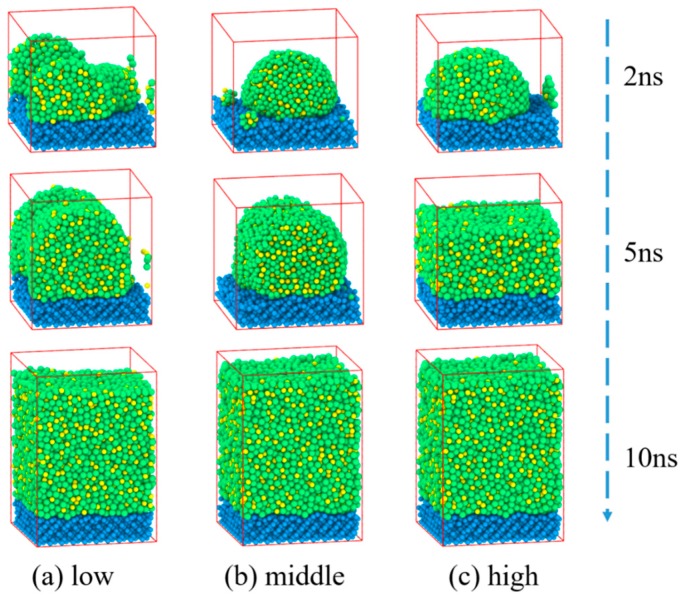
Snapshots of Zr_70_Cu_30_ deposited on Si substrate at 2 ns, 5 ns and 10 ns under different energies. The (**a**), (**b**), and (**c**) show the deposition process under three different energies, and the films grew to approximately 6.0 nm, 6.4 nm, and 6.2 nm thickness respectively.

Deposition processes of Zr_70_Cu_30_ on Si (1 0 0) at 2 ns, 5 ns and 10 ns under three different energies are presented in Figure 4. Initially, small nanoclusters formed on the Si (1 0 0) surface and formed thin films gradually as the number of incoming atoms increases. One can see from Figure 4 that as increasing the mean kinetic energies, the morphology goes through three stages: cluster–cluster–film at 5 ns. At high energy, the thin film was formed before the other two energies. Finally, Zr_70_Cu_30_ films have been formed with thickness ranging from 6.0 to 6.4 nm.

**Figure 5:**
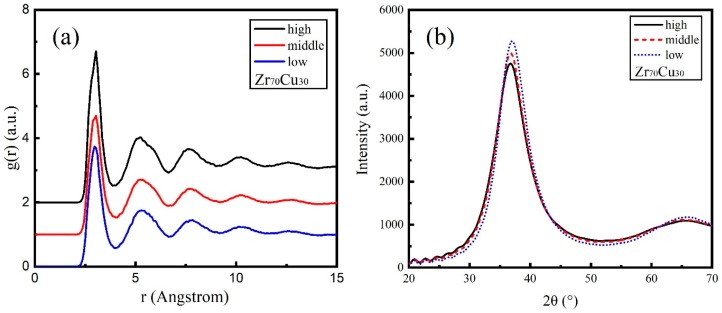
Simulated deposition of Zr_70_Cu_30_ thin films. (**a**) Radial distribution function g(r) of deposited alloy film under different energy conditions, and the abscissa indicates atomic distance r; (**b**) the relationship between X-ray diffraction intensity and diffraction angle 2θ of deposited alloy film under different energy conditions.

The total RDF distributions of Zr_70_Cu_30_ deposited films are displayed in Figure 5 for three deposition energies. The first and second peaks broaden with no splits which shows a typical amorphous characteristic. The amorphous nature of Zr_70_Cu_30_ films under three energies is clearly shown in the snapshots presented in Figure 4, which means the near-eutectic alloy Zr_70_Cu_30_ has a strong GFA.

The simulated XRD intensities versus diffraction angle 2θ for Zr_70_Cu_30_ are illustrated in Figure 5b. Broad diffraction peaks with no split show an amorphous Zr_70_Cu_30_ structure. As the mean kinetic energies increases, the first peak decreases successively and the corresponding diffraction angle 2θ decreases. The main peak diffraction angles for Zr_70_Cu_30_ under three energies are around 37 degrees.

### 3.3. Zr_90_Cu_10_

**Figure 6:**
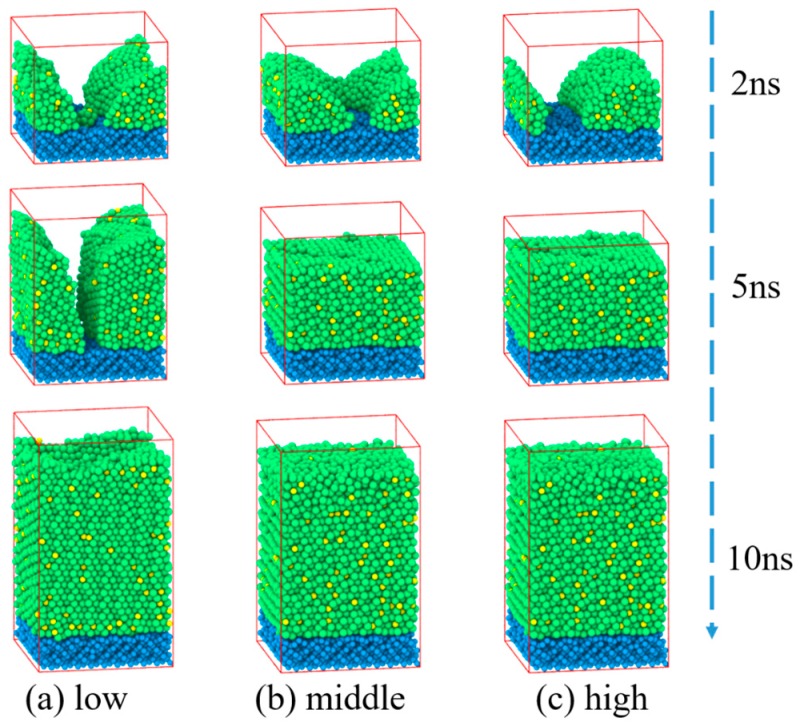
Snapshots of Zr_90_Cu_10_ deposited on Si substrate at 2 ns, 5 ns and 10 ns under different energies. The (**a**), (**b**), and (**c**) show the deposition process under three different energies, and the films grew to approximately 7.1 nm, 7.0 nm, and 7.0 nm thickness respectively.

The deposition processes of the Zr_90_Cu_10_ are displayed in Figure 6. Zr_90_Cu_10_ is deposited as a cluster at 5 ns under low energy, and a film is formed with thickness about 7.1 nm at the end. Under middle and high energies, the deposited atoms grow as films with height around 7.0 nm.

**Figure 7:**
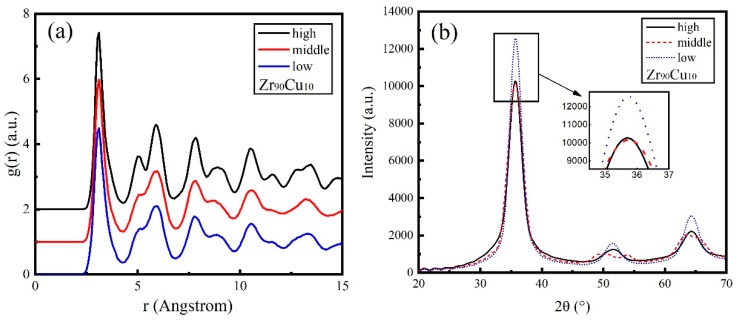
Simulated deposition of Zr_90_Cu_10_ thin films. (**a**) Radial distribution function g(r) of deposited alloy film under different energy conditions, and the abscissa indicates atomic distance r; (**b**) the relationship between X-ray diffraction intensity and diffraction angle 2θ of deposited alloy film under different energy conditions.

The RDF of Zr_90_Cu_10_ under three energies was calculated and is plotted in Figure 7a. For Zr_90_Cu_10_ deposition RDF, four distinct main peaks with a split of second peak arise, showing the well-crystallized structure. The crystallinity under the low energy was better than that of the other two energies according to the peak size and the split degree of second peak, that means the Zr_90_Cu_10_ deposited film shows a better crystal structure under the low energy.

The relationship between XRD intensity and diffraction angle 2θ of Zr_90_Cu_10_ alloy films is presented in Figure 7b for different deposition energies. The XRD patterns exhibit three main peaks at all three cases, and the second peak at the middle energy shows a split. The position of the main XRD peak for Zr_90_Cu_10_ under three energies are between 35 and 36 degrees. Similar to the RDF pattern, the XRD pattern also shows that the three Zr_90_Cu_10_ alloy films have a perfect crystal structure. Among them, the peak of XRD pattern under low energy is bigger, but overall, the difference of XRD pattern under three conditions is not obvious.

**Figure 8:**
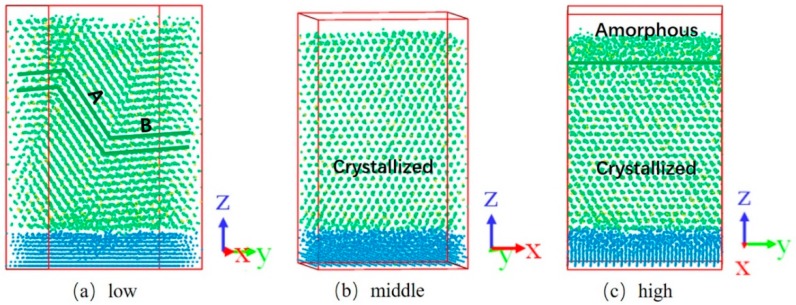
Snapshots of Zr_90_Cu_10_ deposited at three energies. (**a**) The deposited film is a polycrystalline structure with both A and B crystal lattice orientations under low energy conditions; (**b**) the deposited film is a single crystal structure under middle energy conditions; (**c**) the deposited film exhibits a crystal-amorphous structure under high energy conditions.; the green solid line in (**c**) separate the crystalline and amorphous zones.

For deeper understanding of the film growth mechanism, snapshots of Zr_90_Cu_10_ deposited films under different energies are shown in Figure 8. The deposited film is a polycrystalline structure with both A and B crystal orientations under low energy (Figure 8a). At the beginning of the deposition process, small clusters are formed with a random orientation distribution. With time goes on the film growth rates is almost the same, but the growth directions are different under different kinetic energies. This is because that the mean kinetic energy of the deposited atoms is less than the activation energy used for diffusion under low energy, thus forming two cluster structures with different growth directions. Nanostructures with different lattice orientations were formed as the deposition proceeds, that shows that the alloy thin film can be controlled toward a predetermined single crystal or poly-crystal direction by changing the energy condition of Zr_90_Cu_10_. The deposited film has a single crystal structure under middle energy (Figure 8b). While Figure 8c shows a crystalline structure on the bottom and an amorphous structure on the top. The thickness of the amorphous structure is about 1.5 nm.

Based on these simulations, the thickness of films increases when increasing Zr concentrations. This caused by the difference of atomic radius, since the radius of Zr atoms is larger than that of Cu atoms. Comparing the RDF and XRD patterns of Zr_x_Cu_100−x_ (x = 50, 70, 90) deposited films under three energies, it is easy to find that the impact of the elemental composition is bigger than deposition conditions (mean kinetic energy) for GFA of Zr-Cu system. Meanwhile, Zr_50_Cu_50_ and Zr_70_Cu_30_ have stronger ability to form metallic glass, while Zr_90_Cu_10_ tends to form crystalline structure and high energy is good for forming thin films.

### 3.4. Glass Forming Ability

The GFA of an alloy is related to manufacturing conditions and to its own structure properties [27]. The solid-solution formation rules are applicable to characterize the collective behavior of different elements in alloys [29,42]. The parameters Ω and δ of Zr_x_Cu_100−x_ defined for MD simulation are Zr_90_Cu_10_ (0.6686, 6.12%), Zr_80_Cu_20_ (0.5572, 8.33%), Zr_70_Cu_30_ (0.4981, 9.75%), Zr_60_Cu_40_ (0.4607, 10.65%) and Zr_50_Cu_50_ (0.4363, 11.11%), respectively [38].

**Figure 9:**
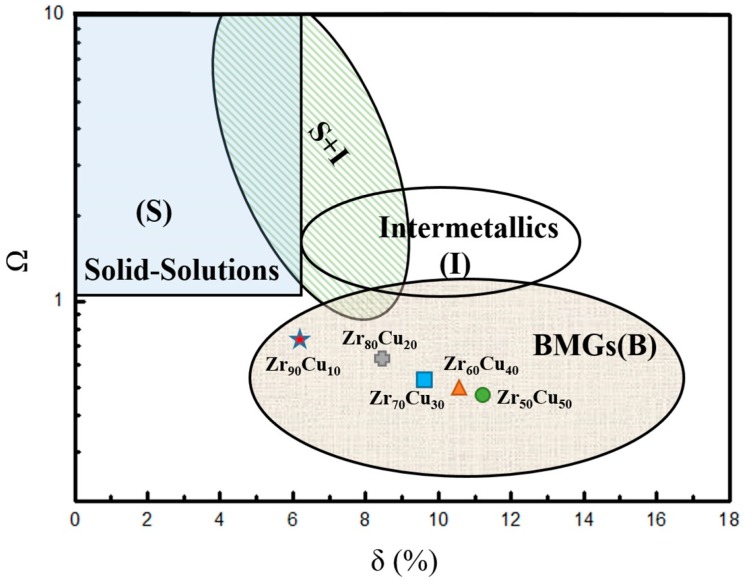
The prediction of phase formation for alloys. The region (S) shows that the alloy can form a solid solution. The alloy in the region (I) mainly consists of intermetallic compounds. The region (S+I) indicate that the solid solution and the intermetallic compound coexist, and alloy forms an amorphous phase metallic glass at the region (B) for bulk metal glasses (BMGs). Ω is the ratio of the average melting temperature timing the mixing entropy and the mixing enthalpy, and δ is the mean square deviation of the atomic size (reproduced from reference [29]).

In the relationship diagram of the Ω and δ (Figure 9), Zr-Cu alloys are located in the area for bulk metallic glass (B). Meanwhile, the Ω and δ values of Zr_50_Cu_50_ and Zr_70_Cu_30_ correspond to the central of BMGs, indicating a strong amorphous forming ability. As increasing the Zr concentration, alloys trend to enter the area of BMGs to solid-solutions. This is consistent with the results of our simulations, that the system prefers to form crystalline structure with high concentration of Zr. For Zr_90_Cu_10_, the thin film structure of polycrystalline, single crystal and crystalline-amorphous composite layer were obtained under low, middle and high energy conditions, which shows that the alloy with high Zr concentration is less likely to form metallic glass under the deposition. Zr_90_Cu_10_ located at the edge of the B area, that is closer to the area of solid-solutions (S) than the others. That theoretically explains our simulation results well.

Three groups of MD simulations revealed the formation of metallic glass microscopically, and the relationship between GFA and parameters (Ω and δ) values. The simulation results which confirmed the correctness of the theory and demonstrated the reliability of MD simulation, is of great significance to predict the thin film structure using magnetron sputtering deposition in MD simulation.

### 3.5. Five-Fold Local Symmetry Analysis.

To gain insights on the GFA at atomic level, we do more atomic structural analysis. The amorphous structure of Zr-Cu alloys thin film was characterized by FFLS, which is one of the most popular methods to describe the nanostructure characteristics of MGs [43,44]. Different atomic clusters may contain different degree of FFLS, which can be expressed by $f_{i}^{k}=n_{i}^{k}/\sum_{k=3,4,5,6}n_{i}^{k}$, where $n_{i}^{k}$ (*k* = 3,4,5,6) is the number of *k*-edged polygon in Voronoi polyhedron type *i*. And the average degree of *k*-fold local symmetry can be expressed by $\sum_{i}f_{i}^{k}\times P_{i}$, where $P_{i}$ is the fraction of *i*. Thus, the degree of FFLS for a polyhedron type can be expressed by $f_{i}^{5}=n_{i}^{5}/\sum_{k=3,4,5,6}n_{i}^{k}$ and the average degree of FFLS can be expressed by $W=\sum_{i}f_{i}^{k}\times P_{i}$.

**Figure 10:**
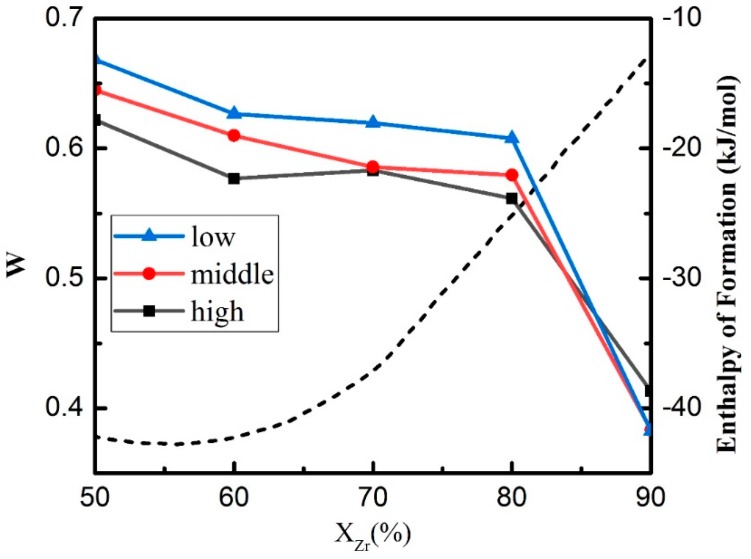
The evolution of five-fold local symmetry in Zr_x_Cu_100−x_. W is the average degree of five-fold local symmetry (FFLS). The component dependence of W in different energy conditions shows similar trend. The dash line is the formation enthalpy of Zr-Cu amorphous alloys from pure amorphous elements at the temperature of 298 K (reproduced from Turchanin [28]).

The typical Voronoi polyhedrons (VPs) with three components are calculated and shown in Table S2 and Figure S1 in the supplementary information. According to the above-mentioned formula about FFLS, we calculate the average degree of FFLS. Solid line in Figure 10 shows the component dependence of the average degree of FFLS, which is denoted as W) under different energies obtained in MD simulations. With increasing Zr concentrations, W decreases gradually. The W of the first four components (x = 50, 60, 70 and 80) are at a relatively high level, especially for Zr_50_Cu_50_. Zr_50_Cu_50_ has the strongest GFA and the largest W value, and exhibits perfect amorphous structure in our simulations.

As shown in Figure 10, the increase of FFLS with decreasing the mean kinetic energy of incoming atoms indicates that FFLS shows energy dependence expect for Zr_90_Cu_10_. The largest W value occurs at the lowest energy shows that reducing the mean kinetic energy of incoming atoms will increase the five-fold local symmetry of the amorphous alloy. The GFA of Zr_90_Cu_10_ is the smallest, and the decrease of W is the most obvious. The difference between Zr_90_Cu_10_ and Zr_x_Cu_100−x_ (x = 50, 60, 70 and 80) is that the maximum W of Zr_90_Cu_10_ appears in high energy conditions. This may be because of the well crystal characteristic exhibited in Zr_90_Cu_10_, and the disorder layer of 1.5 nm appearing on the surface of the film at high energy. At the same time, it shows that the FFLS is not suitable for the description of the long-range order structure.

The component dependence of the formation enthalpy of Zr-Cu amorphous at 298 K is shown in the dash line (Figure 10) [28]. It can be seen that the amorphous formation enthalpy of the Zr-Cu alloy is negative, and the enthalpy of the amorphous formation increases with the increase of Zr concentration when the Zr concentration is greater than 50%. As the value of x increases, its GFA decreases, which is consistent with our simulation result.

Nearly eutectic Zr_70_Cu_30_ films deposited at three energy conditions have amorphous structures shown in our simulations. Combined with its FFLS and Voronoi polyhedral index, it is easy to find that the deposited film has better five-fold local symmetry and the highest content of icosahedron-like clusters under low energy, most of which are Cu-centered. At the same time, using the average degree of FFLS, it is understandable that under low energy the deposited atoms formed film firstly at 5 ns (Figure 4), because it has largest degree of FFLS. On the other hand, as shown in Figure 9, the GFA of five alloys decrease with increasing x, which is coincident with the variation of the five-fold local symmetry that decrease with the x. Our results demonstrate that there is a positive correlation between GFA and the degree of FFLS of Zr-Cu systems.

## 4. Conclusions

We have investigated the correlation between the glass forming ability and the degree of the FFLS in the deposition process of Zr_x_Cu_100−x_ (x = 50, 70, 90) alloy thin films via molecular dynamics simulations. The structural state of alloy films was analyzed by RDF, XRD, Voronoi polyhedron index and FFLS. We find that Zr_90_Cu_10_ films all formed crystals under different energy deposition conditions. There are polycrystalline structure with two crystal orientations under low energy, single crystal under middle energy, and a crystalline-amorphous composite structure under high energy. The formation of crystalline-amorphous structure in Zr_90_Cu_10_ indicates that alloys located at the edge of area B can be prepared into a crystalline-amorphous composite by magnetron sputtering under certain conditions.

The Voronoi polyhedron analysis shows that the greater the number of icosahedral clusters and their deformed structures, the greater the GFA. The Voronoi polyhedron has an important influence on the GFA of Zr-Cu system, and also reveals the relationship between composition and GFA. There is a positive correlation between the degrees of FFLS and GFA. Our results imply that the degrees of FFLS could serve as a measure of the GFA, providing new approach to characterize the GFA for the design of amorphous composite materials. More importantly, our results manifest that sputtering conditions greatly affect the local atomic structures and consequential properties. This result could be extended to other glassy materials as the universality of FFLS for short-range order structure in amorphous. Our study might be useful in optimizing sputtering conditions in real experiments, as well as promising implications in material design of advanced glassy materials.

## Figures and Tables

**Table 1:** The calculated mean kinetic energy for depositions.

Sample of Mean Kinetic Energy	Zr (eV)	Cu (eV)
(a) Low energy group	0.13	0.34
(b) Middle energy group	7.65	6.67
(c) High energy group	12.6	9.61

## Supplementary Materials

The following are available online at http://www.mdpi.com/1996-1944/11/12/2548/s1, Table S1: The four nearest neighbor distances in Zr and Cu bulk crystals, lattice constants for Zr and Cu are respectively $a_{\mathrm{Zr}}$ = 3.23 Å, $c_{\mathrm{Zr}}$ = 5.15 Å and $a_{\mathrm{Cu}}$ = 3.61 Å., Table S2: Number of various Voronoi polyhedrons in Zr_x_Cu100−x (x = 90, 70, 50) under middle energy, Figure S1: Distribution and proportion of typical Voronoi polyhedrons (VPs) in Zr70Cu30 amorphous films under three energies. The left side is FCC-like structure, with ICOS and DICOS in the middle, and other high-content polyhedron on the right.
