# Supplementary material for: Sensitive Five-Fold Local Symmetry to Kinetic Energy of Depositing Atoms in Cu-Zr Thin Film Growth
Source: Materials (Basel). 2018 Dec 14;11(12):2548. doi: 10.3390/ma11122548 (PMC6315649; doi:10.3390/ma11122548)
Supplement: Supplementary file 1 [file materials-11-02548-s001.pdf]

materials MDPI

Supplementary Materials: Sensitive Five-Fold Local Symmetry to Kinetic Energy of Depositing Atoms in Cu-Zr Thin Film Growth

Lu Xie, Haojie An, Qing Peng[2], Qin Qin, Yong Zhang, Xiao Yang and Congjia Su

The Initial Velocity of Incoming Atoms

The initial velocity of the atoms is given by setting the mean kinetic energy which are calculated according to the modification of Thompson formula [24]:

$$f(E) \propto \frac{1-\left(\left(E_{coh}+E\right)/\gamma E_{Ar^+}\right)^{1/2}}{E^2\left(1+E_{coh}/E\right)^3} \tag{1}$$

$$E_F = \left(E-\kappa_B T_g\right)\exp\left[n\ln\left(E_f/E_i\right)\right]+\kappa_B T_g \tag{2}$$

$E_f / E_i = 1-\gamma/2$ is the ratio of energies after and before a collision [25], where $\gamma = 4\frac{m_g m_s}{\left(m_g+m_s\right)^2}$, where m_g and m_s stand for gas atom (Argon here) and sputtered atom (Zr or Cu here) masses respectively, and $n = dp\tau / k_B T_g$, which is the number of collisions that take place in the gas. E is the energy of the sputtered particles as they leave the target, T_g is the sputtering gas temperature, d is the traveled distance, p is the sputtering gas pressure, and τ is the collision cross section assuming hard core interactions. To calculate the energy loss of sputtered atoms with the gas atoms, a Maxwell–Boltzmann (MB) distribution at T_g is fixed for the gas (in our conditions T_g = 300 K). Because we search for the complete distribution of sputtered atoms, we replace $k_B T_B$ by E_g, a particular value in the gas distribution. Thus for each E_g in the MB gas distribution, the energy loss is calculated for a fixed value of the kinetic energy E of a sputtered atom. This is repeated for each E in the Thompson distribution and weighted by the collision probability, which is simply the product of $f(E)$ and the MB distribution at T_g [24].

Table S1. The four nearest neighbor distances in Zr and Cu bulk crystals, lattice constants for Zr and Cu are respectively a_{Zr} = 3.23 Å, c_{Zr} = 5.15 Å and a_{Cu} = 3.61 Å。.

Element	First Neighbor (Å)	Second Neighbor (Å)	Third Neighbor (Å)	Fourth Neighbor (Å)
Zr (hcp)	$a_{Zr} = 3.23$	$\sqrt{2}a_{Zr} = 4.53$	$c_{Zr} = 5.15$	$\sqrt{2}c_{Zr} = 5.57$
Cu (fcc)	$\frac{a_{Cu}}{\sqrt{2}} = 2.55$	$a_{Cu} = 3.61$	$\sqrt{\frac{3}{2}}a_{Cu} = 4.42$	$\sqrt{2}a_{Cu} = 5.10$

Voronoi Polyhedron Analysis

The major Voronoi polyhedral types and numbers of Zr_xCu_{100-x}（x = 90、70、50）thin film at middle energy conditions are listed in Table S2 and Figure S1. There are many Voronoi polyhedrons (VPs) in $Zr_{50}Cu_{50}$ and $Zr_{70}Cu_{30}$ glass films, among which VPs with coordination numbers Z = 12, 13 and 14 are the most abundant, such as <0, 2, 8, 2>, <0, 3, 6, 4> and <0, 2. 8, 4>. There are a large number of regular icosahedrons (ICOS) <0, 0, 12, 0> and distorted icosahedrons (DICOS) <0, 2, 8, 2> and <0, 3,

6, 3>, most of which are centered on Cu. The Cu-centered VPs appear frequently with Z ≤ 12, and the Zr-centered VPs are mainly highly coordinated, among which VPs with Z = 14 (such as <0, 2, 8, 4>, <0, 1, 10, 3> and <0, 3, 6, 5>) are the most abundant, and then VPs with Z = 13 (such as <0, 1, 10, 2>), followed by VPs with Z = 15 (such as <0, 1, 10, 4> and <0, 2, 8, 5>). The $Zr_{90}Cu_{10}$ alloy film has a single crystal structure under middle energy, and its Voronoi polyhedral type mainly concentrates on <0, 4, 4, 4>, <0, 3, 6, 4>, <0, 5, 2, 6> and <0., 4, 4, 6>, while the remaining types accounted for a small percentage.

Table S2. Number of various Voronoi polyhedrons in Zr_xCu_{100-x}（x = 90, 70, 50）under middle energy.

Voronoi index	$Zr_{90}Cu_{10}$			$Zr_{70}Cu_{30}$			$Zr_{50}Cu_{50}$		
	Cu-centered	Zr-centered	Total	Cu-centered	Zr-centered	Total	Cu-centered	Zr-centered	Total
<0, 2, 8, 0>	1	5	6	20	3	23	30	1	31
<0, 3, 6, 1>	17	112	129	21	7	28	31	14	45
<0, 4, 4, 2>	1	7	8	16	4	20	19	11	30
<0, 2, 8, 1>	1	14	15	116	41	157	160	28	188
<0, 3, 6, 2>	9	51	60	47	27	74	31	16	47
<0, 4, 4, 3>	15	99	114	37	17	54	42	13	55
<0, 0, 12, 0>	0	0	0	86	46	132	269	18	287
<0, 2, 8, 2>	3	26	29	202	72	274	321	37	358
<0, 3, 6, 3>	11	48	59	109	63	172	131	46	177
<0, 4, 4, 4>	26	213	239	69	24	93	72	20	92
<0, 3, 7, 2>	0	3	3	10	27	37	26	16	42
<1, 2, 6, 3>	0	9	9	69	47	116	139	17	156
<1, 2, 5, 4>	0	7	7	79	21	100	83	13	96
<0, 4, 5, 3>	1	11	12	9	17	26	8	13	21
<0, 5, 2, 5>	11	15	26	8	8	16	3	3	6
<0, 1, 10, 2>	0	12	12	59	167	226	194	103	297
<0, 3, 6, 4>	30	289	319	216	242	458	280	96	376
<1, 0, 9, 3>	0	1	1	22	39	61	75	17	92
<0, 2, 8, 3>	0	10	10	44	63	107	85	36	121
<0, 4, 4, 5>	15	61	76	36	34	70	59	26	85
<0, 3, 8, 2>	0	1	1	0	7	7	0	3	3
<0, 4, 5, 4>	1	9	10	16	54	70	38	17	55
<0, 4, 6, 3>	0	4	4	1	18	19	3	8	11
<0, 3, 7, 3>	1	4	5	2	36	38	12	25	37
<0, 5, 2, 6>	115	382	497	9	13	22	18	6	24
<0, 2, 8, 4>	0	33	33	40	361	401	74	198	272
<0, 3, 6, 5>	4	72	76	33	220	253	60	120	180
<0, 3, 7, 4>	0	2	2	2	82	84	9	67	76
<0, 0, 12, 2>	0	0	0	1	45	46	3	62	65
<0, 1, 10, 3>	1	0	1	10	174	184	14	145	159
<0, 4, 4, 6>	37	805	842	45	144	189	65	64	129
<0, 1, 10, 4>	0	0	0	0	169	169	1	217	218
<0, 4, 4, 7>	1	98	99	3	89	92	9	58	67
<0, 2, 8, 5>	0	3	3	2	229	231	7	256	263

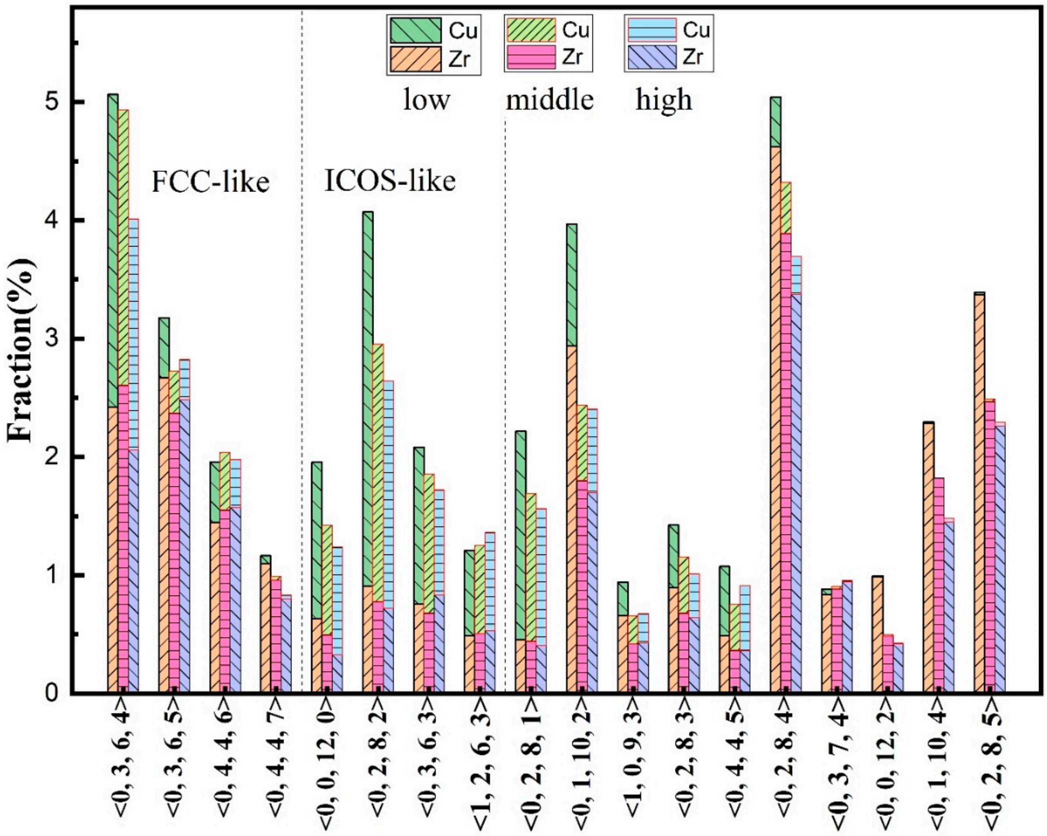

Figure S1. Distribution and proportion of typical Voronoi polyhedrons (VPs) in $Zr_{70}Cu_{30}$ amorphous films under three energies. The left side is FCC-like structure, with ICOS and DICOS in the middle, and other high-content polyhedron on the right.

Figure S1 shows the top 18 representative clusters of near-eutectic $Zr_{70}Cu_{30}$ under different deposition energy conditions. The VPs are divided into three parts. The first part is the Voronoi indices of the distorted fcc polyhedron <0, 3, 6, 4>, <0, 3, 6, 5>, <0, 4, 4, 6> and <0, 4, 4, 7>. The second part is perfect ICOS <0, 0, 12, 0> with Z = 12 and DICOS <0, 2, 8, 2>, <0, 3, 6, 3> and <0, 2, 8, 1 >, and the last part is other highly populated VPs. The fractions of DICOS <0, 2, 8, 2> is higher than the regular ICOS <0, 0, 12, 0>. It can be seen that the formation of polyhedral clusters has a certain energy-dependence. The number of FCC-like clusters is the highest under low energy conditions. Similarly, the fraction of ICOS and DICOS presents the same trend.

© 2018 by the authors. Submitted for possible open access publication under the terms and conditions of the Creative Commons Attribution (CC BY) license (http://creativecommons.org/licenses/by/4.0/).
